# Supplementary material for: Manipulating hyperbolic transient plasmons in a layered semiconductor
Source: Nat Commun. 2024 Jan 24;15:709. doi: 10.1038/s41467-024-44971-3 (PMC10808201; doi:10.1038/s41467-024-44971-3)
Supplement: Supplementary file 1 — Supplementary Information [file 41467_2024_44971_MOESM1_ESM.pdf]

Supplementary Information for

Manipulating Hyperbolic Transient Plasmons in a Layered Semiconductor

Rao Fu[1†], Yusong Qu[2†], Mengfei Xue[3†*], Xinghui Liu[1,4], Shengyao Chen[5], Yongqian Zhao[1,6], Runkun Chen[7], Boxuan Li[1], Hongming Weng[1,8], Qian Liu[2,5*], Qing Dai[2*], Jianing Chen[1,8*]

1. Beijing National Laboratory for Condensed Matter Physics, Institute of Physics, Chinese Academy of Sciences & School of Physical Sciences, University of Chinese Academy of Sciences, Beijing 100190, China.
2. CAS Key Laboratory of Nanophotonic Materials and Devices, National Center for Nanoscience and Technology & School of Nanoscience and Engineering, University of Chinese Academy of Sciences, Beijing 100190, China.
3. Suzhou Laboratory, Suzhou 215100, China.
4. State Key Laboratory of Quantum Optics and Quantum Optics Devices, Institute of Laser Spectroscopy, Collaborative Innovation Center of Extreme Optics, Shanxi University, Taiyuan, Shanxi 030006, China.
5. MOE Key Laboratory of Weak-Light Nonlinear Photonics, TEDA Institute of Applied Physics, School of Physics, Nankai University, Tianjin 300457, China.
6. Wenzhou Institute, University of Chinese Academy of Sciences, Wenzhou 325001, China.
7. State Key Laboratory of Structural Chemistry, Fujian Institute of Research on the Structure of Matter, Chinese Academy of Sciences, Fuzhou, 350002, China.
8. Songshan Lake Materials Laboratory, Dongguan, Guangdong 523808, China.

†These authors contributed equally: Rao Fu, Yusong Qu, and Mengfei Xue.

*E-mails: xuemf@szlab.ac.cn; liuq@nanoctr.cn; daiq@nanoctr.cn; jnchen@iphy.ac.cn;

Supplementary Note 1: TEM and Raman spectra

The BP crystals tend to be cleaved along the zigzag direction, resulting in rectangular BP slabs with two long straight edges parallel to the zigzag direction, as depicted in Supplementary Figure 1a. The atomic structure of BP in Supplementary Figure 1b shows that the lattice-constant a along the zigzag direction is 3.316 Å, and the lattice-constant c along the armchair direction is 4.389 Å.[1] Supplementary Figure 1c-d shows the TEM images of a black phosphorus (BP) ribbon. To verify that the long straight edge of the BP ribbon is parallel to the zigzag direction, we chose the red box region in Supplementary Figure 1c to zoom in to identify the atomic arrangement of the BP ribbon. The high-resolution TEM image in Supplementary Figure 1d clearly shows that the interval period of phosphorus atoms is 3.46 Å along the direction parallel to the long straight edge, and the interval period of phosphorus atoms is 4.53 Å along the direction perpendicular to the long straight edge. Accordingly, the zigzag (armchair) direction is parallel (perpendicular) to the long straight edge. The presence of an amorphous structure at the BP edge in Supplementary Figure 1d is the slight oxidation.

We also performed Raman spectroscopy to corroborate the atomic arrangement given by the high-resolution TEM image in Supplementary Figure 1d. The laser and collected light polarization were fixed to be parallel to each other in the measurement configuration. Supplementary Figure 1e is the Raman spectrum of a rectangular BP ribbon, from which three vibrational modes A_g^1 (359.5 cm^{-1}), B_{2g} (436.8 cm^{-1}), and A_g^2 (464.0 cm^{-1}) can be identified. Supplementary Figure 1f shows the intensity of A_g^2 at different angles between the BP's long straight edge and the laser polarization. Due to the BP's low symmetry, the angular dependence A_g^2 can be used to identify the lattice orientations[2]. The angular-dependent A_g^2 exhibits a cross-like shape with a long axis and a short axis; the short axis is parallel to the zigzag direction, and the long axis is parallel to the armchair direction. Therefore, the Raman spectrum and TEM images confirm that the zigzag direction is parallel to the long straight edge of the BP ribbon.

Supplementary Note 2: Dynamics of BP plasmon

The time-resolved amplitude $s_{3,BP}/s_{3,Si}$ and phase $\phi_{3,BP}-\phi_{3,Si}$ spectra in Supplementary Figure 2a-b display plasmon dynamics in the frequency domain from τ = 0 – 30 ps. Using the bi-exponential fitting, two different time constants are achieved in the relaxation of screened plasmon frequency of the z-direction $\omega_{p,z}^{*} = \frac{\omega_{p,z}}{\sqrt{\varepsilon_{\infty,z}}}$ (Supplementary Figure 2c). The fast decay with a τ_1 = 4

ps time constant is consistent with the survival time of the propagating polariton in Fig. 4a. And this 4 ps rapid decay is attributed to the trapping of carriers by the surface defects[3]; the longer decay with a τ_2 = 17 ps time constant reflects the Auger-enhanced bulk carrier recombination at high carrier density[4].

Supplementary Note 3: Excitation and optical emission paths of polariton fringes

Supplementary Figure 3a shows, from top to bottom, three different optical paths of polariton fringes, that is, tip-launching + edge-emission (strength A_1), edge-launching + tip-emission (strength $A_2|sin\,\theta|$), and edge-launching + secondary-tip-launching + edge-emission (strength $A_3|sin\,\theta|$) routes. Both A_1 and $A_2|sin\,\theta|$propagate a one-trip between the edge and the tip, and the corresponding period of fringes is λ_p. The $A_3|sin\,\theta|$ propagates a round-trip between the edge and the tip, and the corresponding period of fringes is $\lambda_p/2$. Supplementary Figure 3b shows the near-field fringes from θ = 0° to 328°. The short-period fringes dominate at θ = 90° and 270°, and the long-period fringes dominate at θ = 0° and 180°. To clarify the alternating domination of the long-period and short-period fringes at different θ, we performed Fourier transforms (FT) on the fringes in Supplementary Figure 3b to extract the magnitude ratio I_{short}/I_{long} of short/long-period fringes (FT results at θ = 0° and 90° in Fig. 2d). We used the $\frac{I_{short}}{I_{long}} \propto \frac{|sin\,\theta|}{1-a|sin\,\theta|}$ to fit the I_{short}/I_{long} ratio, the denominator in this fitting formula represents the destructive interference between A_1 and $A_2|sin\theta|$, and the numerator represents the strength of $A_3|sin\,\theta|$. The fitting results in Supplementary Figure 3c agree well with the experimental data, verifying the correctness of the three optical paths of polariton fringes in Supplementary Figure 3a. Supplementary Figure 3d shows the simulated results of the edge-launching polariton at θ = 90°, 30°, 5°. As the laser polarization is perpendicular to the edge at θ = 90°, the optical field drives the carriers to form a collective oscillation perpendicular to the edge. Thus, at θ = 90°, the edge as an optical antenna can effectively launch the TM mode polariton[5].

Supplementary Note 4: Thickness-dependent polariton wavelength

Supplementary Figure 4a shows the 5-layer structure in the main text (air/5-nm-thick P_xO_y/120-nm-thick high doping BP/intrinsic BP/Si), the dielectric constant of P_xO_y and intrinsic BP is ε_{PxOy} = 27,[6] $\varepsilon_{\infty,x}$ = 18, $\varepsilon_{\infty,y}$ = 14 and $\varepsilon_{\infty,z}$ = 9.75, respectively. Since the band gap of the P_xO_y

layer is 7.2 eV, which is far larger than pump energy hv = 0.8 eV, we do not need to consider the impact of the P_xO_y layer on the pump light[7]. The carrier density distribution in the BP slabs was simplified to a highly doped surface charge layer with a thickness of 120 nm and a beneath low-doping layer in Supplementary Figure 4a. The excellent agreement between the experimental and simulated results in Fig. 3b–f demonstrates that this simplification can be well used for the polariton mode analysis. We also simulated polariton propagation in uniformly doped BP to reveal the hyperbolic volume mode. We adopted the four-layer structure (air/5-nm-thick P_xO_y/high doping BP/Si) in Supplementary Figure 4b. Supplementary Figure 4c shows the real part of z component electric field distribution Re(E_z) at v/c = 950 cm^{-1} of several uniformly doped BP slabs with the BP thickness d increases from 100 nm to 300 nm (the dielectric tensor at v/c = 950 cm^{-1} of BP is in Fig. 1d). As expected, the polariton wavelength of the 300 nm (200 nm) thick BP is three times (two times) that of 100 nm thick BP. The analytical formula for thickness-dependent polariton wavelength at a fixed frequency can be written as (fundamental mode case)[8]:

$$q = -\frac{\psi}{d}\left[tan^{-1}\left(\frac{\varepsilon_{air}}{\varepsilon_x \psi}\right) + tan^{-1}\left(\frac{\varepsilon_{Si}}{\varepsilon_x \psi}\right)\right], \psi = \frac{\sqrt{\varepsilon_z}}{i\sqrt{\varepsilon_x}}$$

Here, ε_{air} = 1 (ε_{si} = 11.9) is the dielectric constant of the air (Si), the polariton wavelength $\lambda_p = \frac{2\pi}{\mathrm{Re}(q)}$, ε_x (ε_z) is the dielectric elements in the armchair (z) direction of the BP at v/c = 950 cm^{-1} (We ignored the thin P_xO_y layer that does not affect the trend of wavelength variation with thickness). Supplementary Figure 4d shows that the wavelength of bulk hyperbolic polaritons increases linearly with increasing BP thickness, confirming the volume polariton mode. Furthermore, in Supplementary Figure 4e, we simulated the dipole excited polaritons in a semi-infinite BP, from which the directional rays of the volume hyperbolic polaritons can be seen. Here, we only show the polariton propagating along the armchair direction. The zigzag direction also has the positive dielectric element. The thickness-dependent polariton wavelength of the volume mode propagating along the zigzag direction is similar to that of the armchair direction. Supplementary Figure 4f shows the simulated propagation mode of Re(E_z) based on pump light wavelength (1560 nm). Using the approximated refractive index of BP (set as $\varepsilon_{x, y, z}$ = 10.3+0.1i, 8.6+0.17i, 10.8+2.67i) and Si (ε_{Si} = 12) at pump light wavelength[9,10], the electromagnetic field could not exhibit as a waveguide mode in BP and instead diffuses into Si quickly. In this work, a

Ge filter (long pass) in the detection light path blocks the pump light in front of the detector in measurement to avoid the possible influence of the pump light.

Supplementary Note 5: Hyperspectral images and plasmonic dispersion behavior

Hyperspectral line-scan imaging was conducted to categorize fringes in the near-field images into specific transient hyperbolic plasmon modes. This imaging technique spatially records the spectrum of each point along a scanned line in a positional sequence, allowing for straightforward mapping of plasmonic dispersions[11-13]. As shown in Supplementary Figure 5a-b, the hyperspectral images of transient hyperbolic plasmons propagating along the armchair and zigzag directions were obtained at τ = 200 fs, respectively. The hyperspectral line scans reveal the periodic nature of the polariton fringes. One-dimensional Fourier transforms in Supplementary Figure 5c and Supplementary Figure 5d convert the line-scan spectra in Supplementary Figure 5a and Supplementary Figure 5b into frequency-momentum diagrams, respectively. It is important to note that the wavevector axis in Supplementary Figure 5c has been divided by a factor of 2 due to the fringe period being $\lambda_p/2$ in Supplementary Figure 5a[12]. Supplementary Figure 5a-d shows that measured transient plasmon modes are narrowly confined within the probing bandwidth, as the oppositely signed dielectric elements only occupy a small portion of the entire probing bandwidth in Fig. 1c. The confined in-plane momentum (q) region of the transient plasmon modes in Supplementary Figure 5e-f is due to the scattering of transient plasmons from A_1 and $A_3|sin\,\theta|$ paths at the BP edge into free-space light. This scattered light is confined around a fixed q, similar to the bell-shaped momentum bandwidth of an AFM tip-excited evanescent field[14]. As a result, the measured in-plane momenta of hyperbolic transient plasmons are confined in a narrow momentum span, as shown by the dashed lines in Supplementary Figure 5c-f[12].

Supplementary Note 6: The behavior of polaritons under different pump fluence

The carrier density in BP increases with the increase of pump fluence. Supplementary Figure 6a-b shows the near-field amplitude spectra $s_{3,BP}/s_{3,Si}$ and phase spectra $\phi_{3,BP}-\phi_{3,Si}$ with the pump fluences increasing from 0 mJ/cm^2 to 0.42 mJ/cm^2. The broad peaks in both the amplitude and phase spectra show a blue shift as the pump fluence increases, indicating an increase in carrier density. When the pump fluence reaches 0.25 mJ/cm^2, the electronic state filling reduces the optical absorption efficiency. When the pump fluence is larger than 0.42 mJ/cm^2, the photo-

induced carrier density reaches saturation and the blue shift of plasmon frequency stops[15]. Supplementary Figure 6b-c are polariton fringes with the pump fluences increasing from 0 mJ/cm^2 to 0.67 mJ/cm^2. Consistent with Fig. 4, the polariton's near-field contrast increases with the carrier density increase. The localized edge mode emerges under 0.16 mJ/cm^2 pump fluence due to the edge's conductivity change and charge enrichment. The localized edge mode is observed under 0.25 mJ/cm^2, while the propagating mode exhibits a similar near-field contrast under 0.33 mJ/cm^2. In Supplementary Figure 6d, we calculated the imaginary part of the Fresnel reflection coefficient of the five-layer system at different plasmon frequencies. As the plasmon frequency increases, the proportion of polariton frequency regions in the probe bandwidth increases, resulting in an improved fringe contrast in Supplementary Figure 6b. The polariton wave vectors in Supplementary Figure 6c have been marked as cyan stars to show the center of polariton frequency.

Supplementary Note 7: Dynamics of polaritons propagating along the zigzag direction

Consistent with the dynamics of the polariton propagating along the armchair direction in Fig. 4. The survival time of polariton propagating along the zigzag direction is also about 6 ps. Nevertheless, since the lattice combination along the zigzag direction is sturdier than that in the armchair direction, the crystals are challenging to cleave in the armchair direction, resulting in the poor quality of the armchair edge. The armchair edge's poor quality increases the density of the surface defect states, which traps the carriers and weakens the charge enrichment at the edge. Hence, no localized edge states are observed in Supplementary Figure 7.

Supplementary Note 8: Photo-induced plasmonic ring of BP/gold disk stacked structure

In Fig .1h and Supplementary Figure 8, BP and gold disk thicknesses are 310 nm and 100 nm, respectively. The hyperbolic IFCs allow the gold disk's edge to launch conical-shaped energy rays, producing two bright rings separated by a dark ring above the round gold disk's edge. Therefore, in Supplementary Figure 8a, the near-field amplitude image under 0.5 mJ/cm^2 pump illuminated clearly shows a dark ring sandwiched by two bright rings above the gold disk. In Supplementary Figure 8b, the pristine BP's near-field image does not show particular optical features only with probe light illumination. In Supplementary Figure 8c, we set τ = -2 ps under 0.5 mJ/cm^2 pumping light so that the probe shines the BP before the pump comes. In this case, the probed BP is also pristine, and neither near-field amplitude image exhibits polaritonic rings. Therefore, the pump-

injected non-equilibrium carriers are crucial to launch BP's hyperbolic plasmons. Supplementary Figure 8d shows the radial line profiles of Supplementary Figure 8a-b, along the same direction of the dashed arrow in Supplementary Figure 8b. The profile line extracted at 0.5 mJ/cm^2 pumping power exhibits the polariton fringe while vanishing at 0 mJ/cm^2 pumping condition. The vertical black dashed line in Supplementary Figure 8d marked the position of the edge of the gold disk. The topography image in Supplementary Figure 8e shows that, after BP coverage, the region where the gold disk below is slightly arched.

Supplementary Note 9: Plasmons of different thin BP slabs

In Supplementary Figure 9a-d, we selected four different BP slabs with thicknesses from 21 – 164 nm to explore the thickness-related polariton fringes. In Supplementary Figure 9e-h, we found that the photo-induced plasmon fringes are only apparent in the BP slabs thicker than 102 nm, and the fringe contrast increases significantly with the increase in BP's thickness.

As the thickness of BP decreases, the fringe spacing in Supplementary Figure 9g (102 nm thick BP) reduces by about 13% compared with Supplementary Figure 9h (equivalent to 120 nm thick BP), which shows the BP's hyperbolic transient plasmon fringe spacing almost linearly decreases with the reduction in BP thickness, similar to the case of hBN phonon polaritons[8]. The cause of fringe vanishing in thin BP flakes may be twofold. One is that the oxidization rate of thin BPs becomes higher[16], leading to an increasing plasmon propagation loss due to impurities scattering[17]. The thinner the flake is, the stronger the oxidization effects. On the other hand, thin BP flakes exhibit a relatively weaker plasmonic response, leading to a decrease in the near-field amplitude signal of plasmon. Similar fringe contrast deduction with flake thickness also occurs in typical hyperbolic materials such as hBN and MoO_3, where the fringe contrast of polaritons decreases rapidly with decreasing thickness[18,19].

The thin BP samples degrade more rapidly in the atmospheric environment and do not last a long measurement time, which is essential for dynamic near-field measurements. Therefore, we prefer thick samples exceeding 200 nm for studying the non-equilibrium plasmons in BP.

Supplementary Note 10: Hyperbolic plasmons in BP covered with a 10-nm-thick hBN protecting layer

In Supplementary Figure 10a, we covered BP with a 10-nm-thick hBN. The spectral dip

observed near 800 cm^{-1} in Supplementary Figure 10c corresponds to the absorption of hBN[20]. In Supplementary Figure 10b, we also observed BP's photo-induced plasmon fringes. This photo-induced plasmon fringe spacing is consistent with the results obtained from bare BP in the main text.

In principle, hBN could protect BP from degradation. However, in Supplementary Figure 1a, the size of the BP is on the order of hundreds of microns. To ensure effective near-field detection of BP's plasmon by the s-SNOM tip, we couldn't use a thick hBN protection layer. Our mechanically exfoliated 10-nm-thick hBN layer could only cover a small portion of the BP, leaving most of the BP exposed to the atmosphere and susceptible to degradation. Unfortunately, the degradation could extend to the regions covered by hBN, rendering hBN's protective function ineffective.

Supplementary Note 11: Effects of BP's degradation on hyperbolic plasmons

We performed near-field imaging on the same BP with different degradation levels. Supplementary Figure 11a-b was captured from freshly exfoliated BP. Supplementary Figure 11c–d was acquired from BP that had been exposed to atmospheric conditions for three days. Upon close examination of the topography in Supplementary Figure 11c, we observe the presence of point-like protrusions. This characteristic indicates the typical degradation of BP.

The dark spots in Supplementary Figure 11d also reveal the evident impact of this degradation. It's worth noting that the BP's hyperbolic plasmon is a bulk mode, similar to the phonon polariton in hBN[21]. Therefore, the electric field of plasmons is primarily confined inside the BP (Fig. 3e-f), which means that the presence of a surface degradation layer, although visually apparent, we can still observe the faint BP's plasmonic modes in Supplementary Figure 11d.

Supplementary Note 12: Monochromatic near-field amplitude images of the pristine BP

Due to the low initial carrier density, the pristine BP does not support hyperbolic plasmons. We performed monochromatic near-field imaging on a 205-nm-thick BP slab using a quantum cascade laser (DRS Daylight MIRcat). The tuning range of the laser frequency covers 940 – 1160 cm^{-1}. Supplementary Figure 12b-i shows the monochromatic near-field imaging under different frequencies, where the BP exhibits uniform near-field signal from 940 – 1160 cm^{-1}. Supplementary Figure 12j shows the near-field profiles from the white dashed line in Supplementary Figure 12b.

The near-field profiles do not show plasmonic fringe from 940 – 1160 cm^{-1}. Another approach to probing BP plasmons is to measure with a Thz laser that might match the pristine BP's low doping conditions.

A pump-probe investigation scheme is necessary to generate sufficient carriers in BP and explore its plasmonic properties. In principle, a pumping laser with photon energy higher than the bandgap is adequate to generate free carriers (Fig. 1b). For the plasmon frequency $\omega_p \propto \sqrt{n}$, the tunable frequency range of ω_p is determined by the maximum n, which depends on the density state of the electron at the conduction band. Hence, in principle, the saturation electron concentration and the up-limit of plasmon frequency can be elevated through high photon energy pumping excitation.

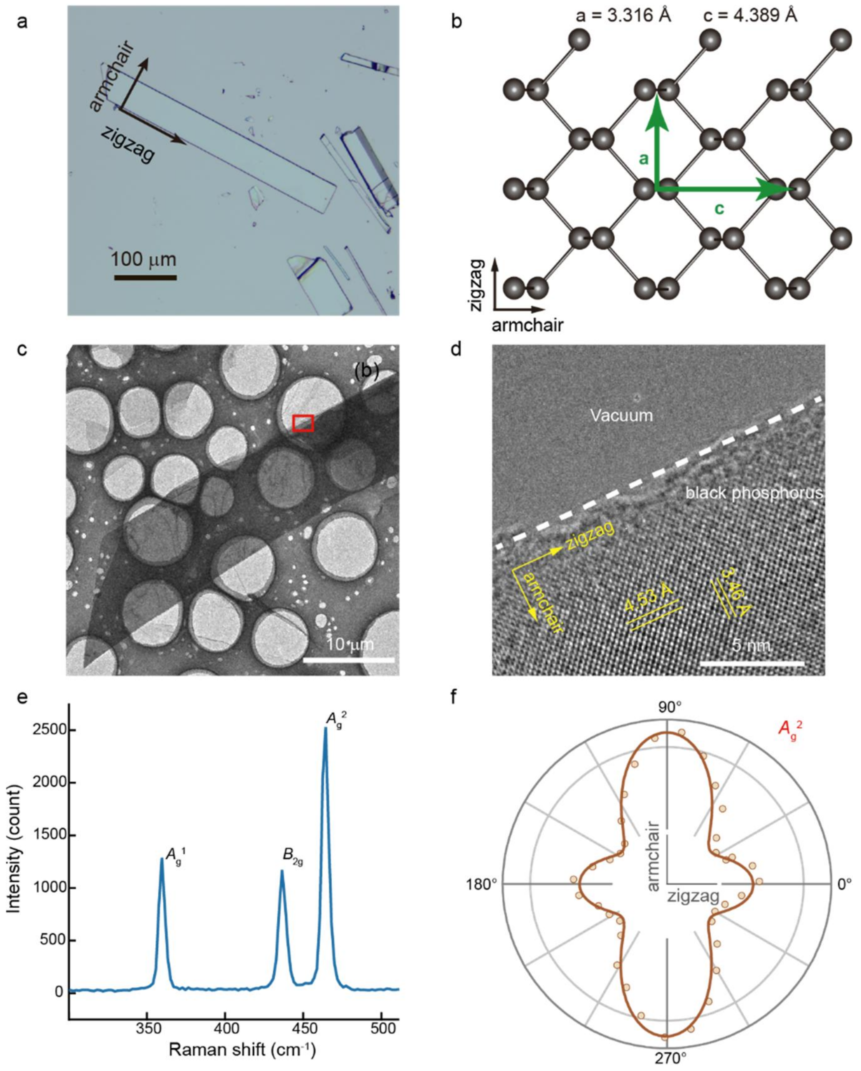

Supplementary Figure 1. TEM and Raman spectra of rectangular BP ribbons. a) The optical image of rectangular-shaped BP slabs. **b)** The atomic configuration of the BP. **c)** The transmission electron microscopy (TEM), and **d)** the high-resolution TEM image taken from the red rectangle region in **(c)**. **e)** Raman spectrum of the BP slabs. The wavelength of the excitation laser is 532 nm. The excitation and collected light polarization are fixed to be parallel. **f)** The angular-dependent intensity for the A_g^2 mode. The angle in the polar coordinate is the angle between the laser polarization direction and the long straight edge of the BP.

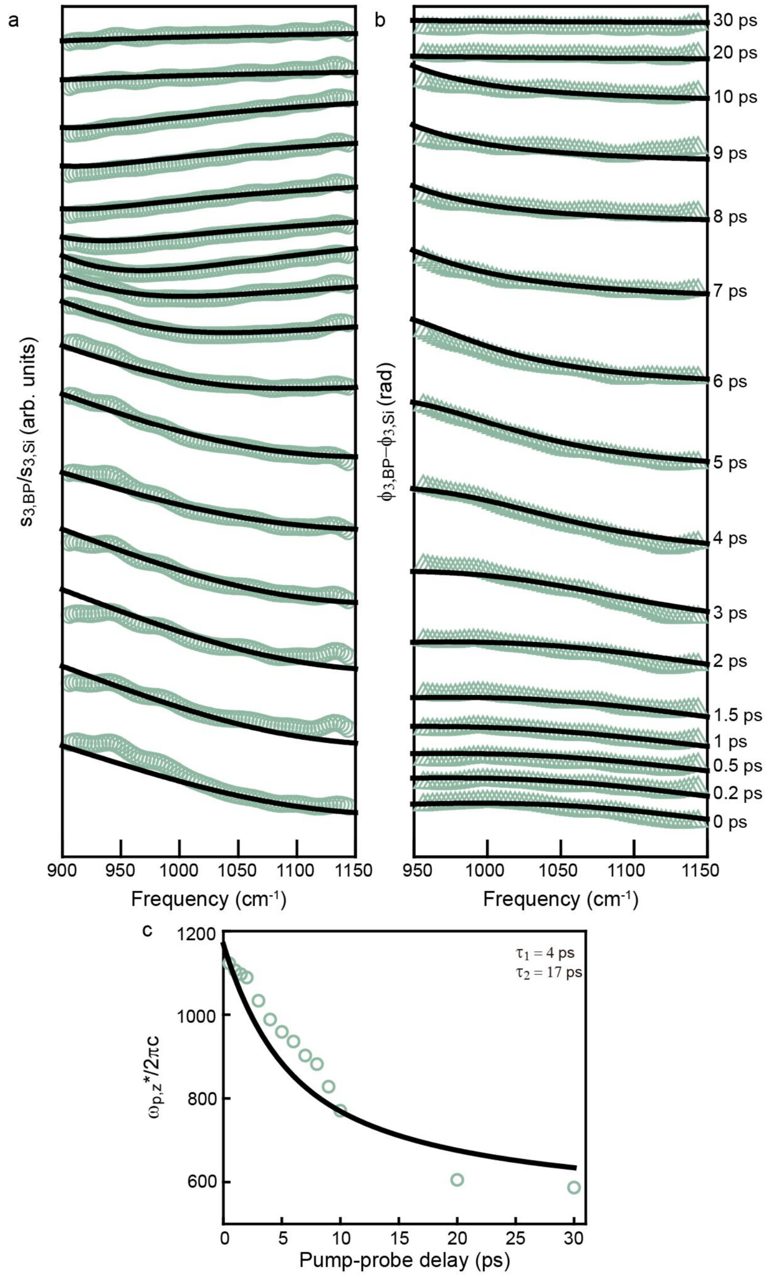

Supplementary Figure 2. Dynamics of the BP plasmon. **a) – b)** Transient near-field amplitude spectra $s_{3,BP}/s_{3,Si}$ **(a)** and phase spectra $\phi_{3,BP}-\phi_{3,Si}$ **(b)** with 0.5 mJ/cm^2 pump fluence. From top to bottom, the pump-probe delay τ is decreased. Opened circles (triangles) represent the experimental data, and solid lines fit the results using the dipole model. Traces are vertically offset for clarity. **c)** Time-evolved screened plasmonic frequency of the z-direction $\omega_{p,z}^*$ presented by the bi-exponential fitting.

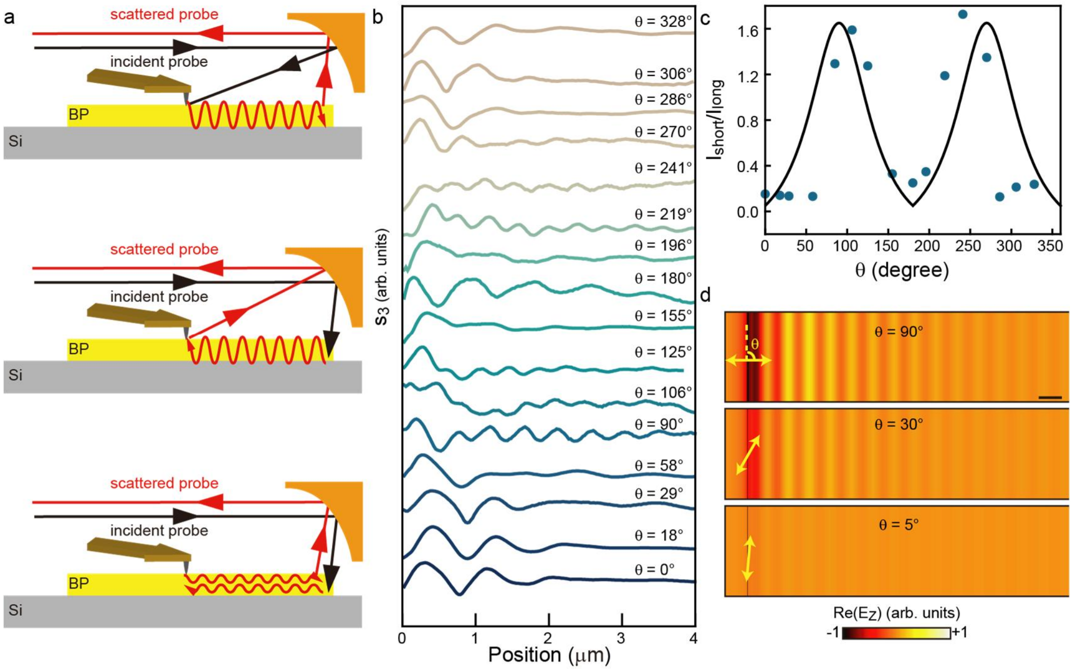

Supplementary Figure 3. Angular-dependent near-filed polariton fringes. a) Three optical paths of the measured near-field fringes. **b)** Polariton fringes at different θ. **c)** The magnitude ratio I_{short}/I_{long} of short/long-period fringe extracted from the Fourier transform of **(b)**. Solid lines are fitting results considering the three possible optical paths in **(a)**. **d)** Simulated near-field distribution Re(E_z) of edge excited polariton mode at v/c = 950 cm^{-1} for θ = 90°, 30°, 5°. The yellow double-arrow is parallel to the direction of the laser polarization—scale bar: 1 μm.

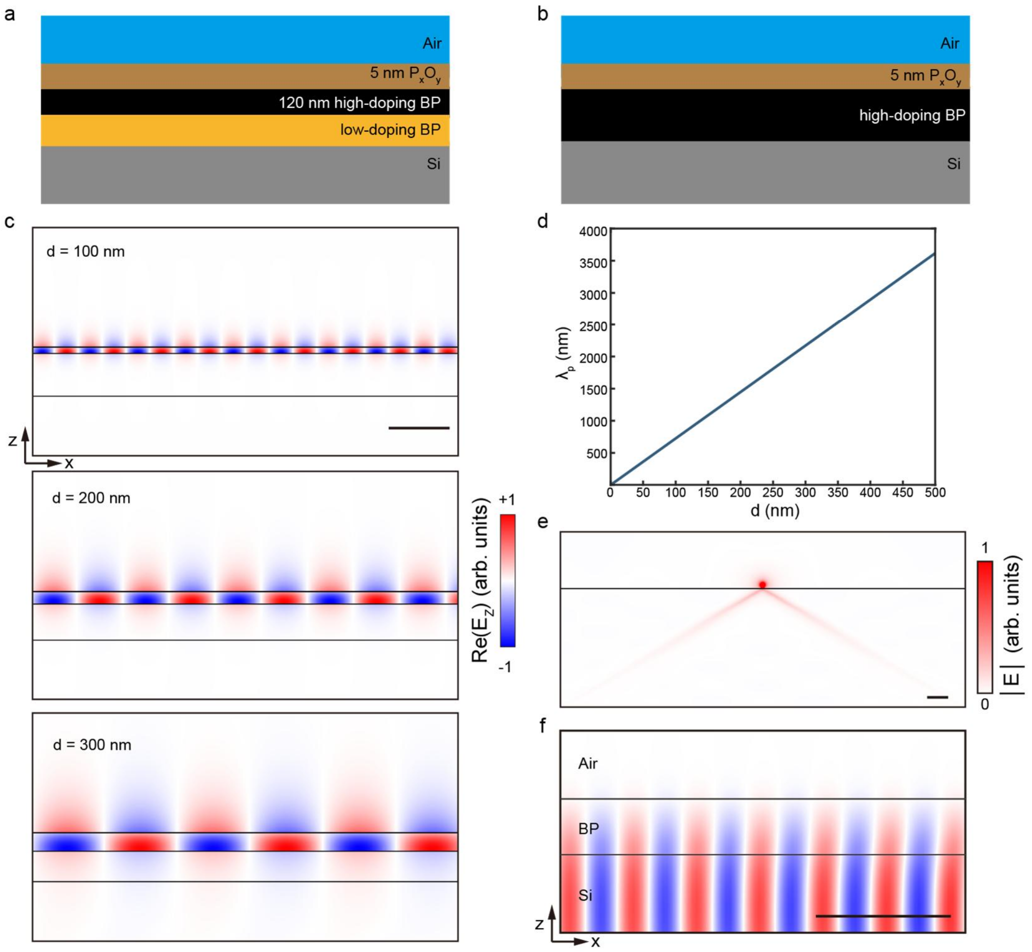

Supplementary Figure 4. Volume hyperbolic polariton in BP slabs. a) Schematic of the five-layer stacking structure in the main text. **b)** Schematic of the four-layer stacking structure. **c)** Simulated real part of z component electric field distribution Re(E_z) of volume hyperbolic polaritons at v/c = 950 cm^{-1} with the uniformly doped BP thickness = 100 nm, 200 nm, and 300 nm, scale bar: 1 μm. **d)** Theoretical calculation of the thickness-dependent polaritons wavelength. **e)** The simulated result of electric field distribution $|E|$ of polaritons in a uniformly doped semi-infinitely thick BP. **(c)** – **(e)** are the simulated/calculated results of the polaritons propagating along the armchair direction. **f)** Simulated near-field distribution Re(E_z) of BP based on pump light wavelength (1560 nm). Scale bar: 1 μm.

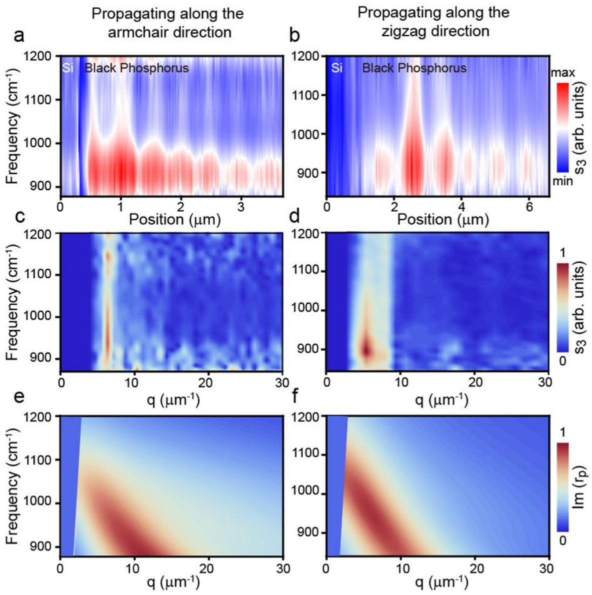

Supplementary Figure 5 Transient plasmon dispersion analysis. a) − b) Hyperspectral line scans along the armchair direction **(a)** (BP thickness 275 nm) and the zigzag direction **(b)** (BP thickness 700 nm) at $\tau = 200$ fs. The polaritons are in a $\nu/c = 850 – 1000\ \mathrm{cm}^{-1}$ narrow bandwidth. **c) − d)** Fourier transformation amplitude spectra of hyperspectral line scans in **(a)** and **(b)**, respectively. The wavevector axis in **(c)** has been divided by a factor of 2 because the fringe period is $\lambda_p/2$ in **(a)**. **e) − f)** Theoretical dispersions of the polariton mode propagating along the armchair **(e)** and the zigzag **(f)** direction calculated by the imaginary part of the Fresnel reflection coefficient $Im(r_p)$, respectively.

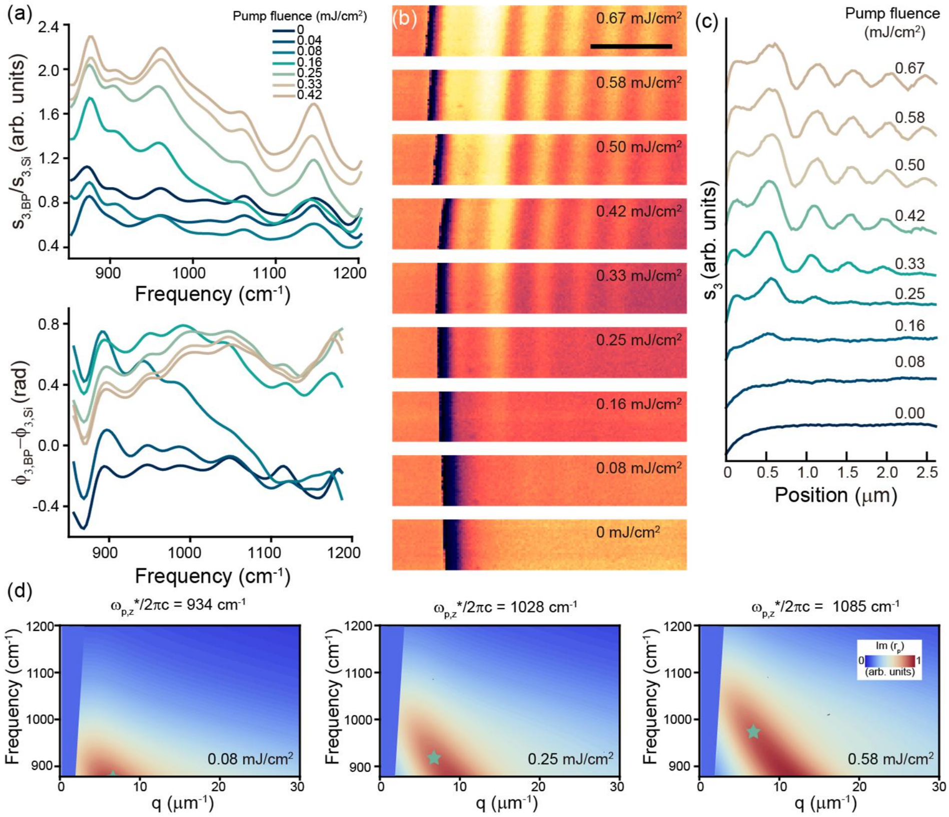

Supplementary Figure 6. Pump fluence-dependent polariton fringes. **a)** Normalized 3rd harmonic near-field amplitude spectra $s_{3,BP}/s_{3,Si}$ (top) and phase spectra $\phi_{3,BP}-\phi_{3,Si}$ (bottom) with the pump fluence increases from 0 mJ/cm^2 to 0.42 mJ/cm^2. **b) – c)** Polariton fringes with the pump fluence increase from 0 mJ/cm^2 to 0.67 mJ/cm^2. The time delay for **(b)** and **(c)** is $\tau = 200$ fs, scale bar: 1 μm. The edge is parallel to the zigzag direction. The BP thickness is 275 nm. **d)** The theoretical dispersion of the polariton mode propagating along the armchair direction is calculated by the imaginary part of the Fresnel reflection coefficient $Im(r_p)$. The star marks show the plasmon wavevectors of different pump fluence in **(c)**. From left to right: $\frac{\omega^*_{p,z}}{2\pi}$ = 934 cm^{-1}, 1028 cm^{-1}, and 1085 cm^{-1} corresponding to pump fluence: 0.08 mJ/cm^2, 0.25 mJ/cm^2, and 0.58 mJ/cm^2.

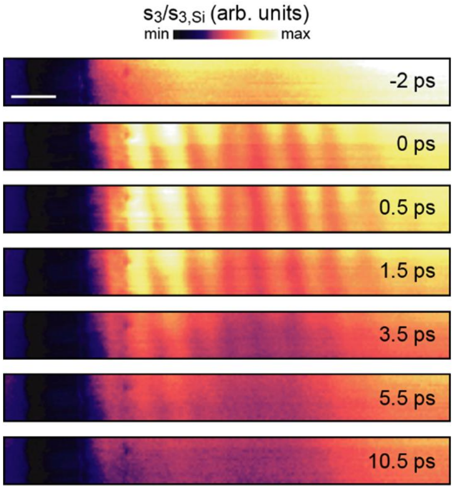

Supplementary Figure 7. Normalized near-field amplitude images $s_3/s_{3,Si}$ of a BP slab (160 nm thickness) for seven different delay times τ, the edge is parallel to the armchair direction, scale bar: 1 μm.

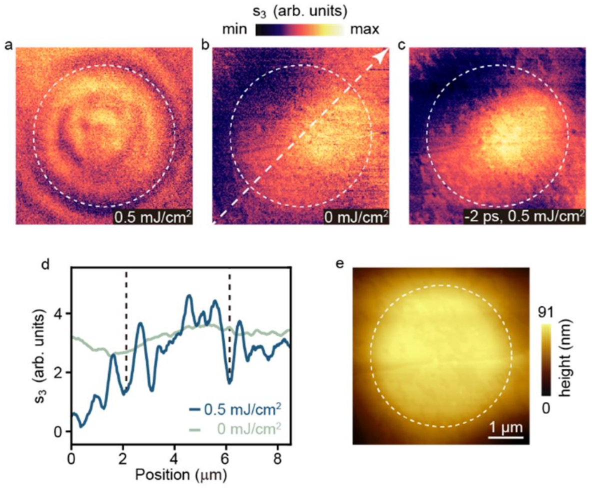

Supplementary Figure 8. Photo-induced plasmonic ring of BP/gold disk stacked structure. a) - c) Near-field amplitude imaging with 0.5 mJ/cm^2 pump fluence **(a)** and 0 mJ/cm^2 pump fluence **(b)** and 0.5 mJ/cm^2 pump fluence at -2 ps **(c)**. **d)** Radial line profiles of **(a)** and **(b)** along the dashed arrow in **(b)**. The dashed line in **(d)** represents the position of the edge of the gold disk. **e)** The topography of BP/gold stacked structure.

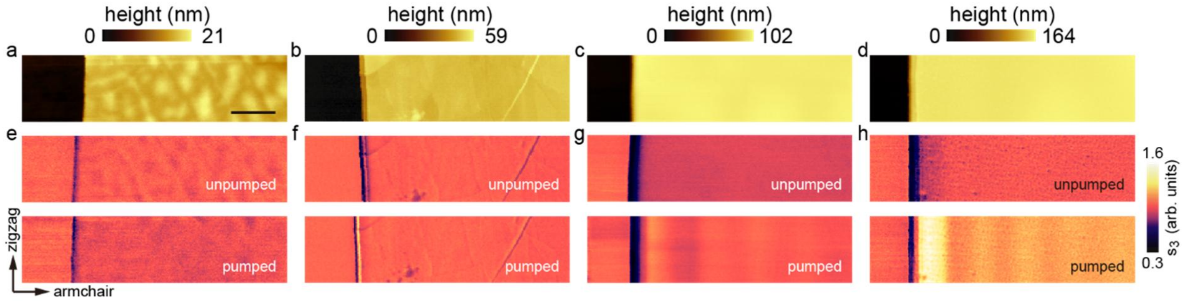

Supplementary Figure 9. Plasmons of different thin BP slabs. a) – d) Topography of BPs with a thickness of 21 – 164 nm. Scale bar: 1 µm. **e) – h)** Near-field amplitude images of BP with a thickness of 21 – 164 nm. The frequency region of the probe is ν/c = 850 – 1200 cm^{-1}.

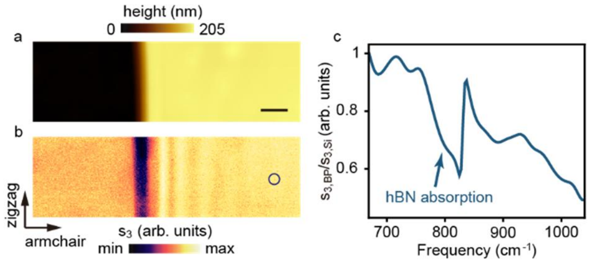

Supplementary Figure 10. Hyperbolic plasmons in BP covered with a 10-nm-thick hBN protecting layer. **a)** The topography image of the hBN-protected BP. Scale bar: 1 µm. **b)** The Near-field amplitude image of hBN-protected BP. The frequency region of the probe is v/c = 850 – 1200 cm^{-1}. **c)** Nano-FTIR of hBN protected BP from the black circle in **(b)**.

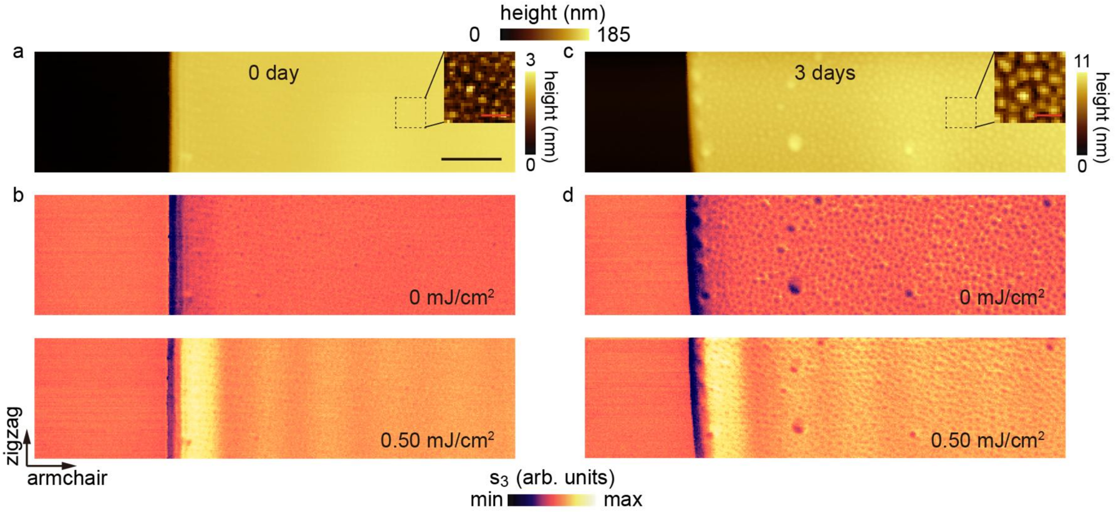

Supplementary Figure 11. Effects of BP's degradation on hyperbolic plasmons. a) – b) The topography **(a)** and the near-field amplitude image **(b)** of the freshly exfoliated BP. Scale bar: 1 μm. The frequency region of the probe is v/c = 850 – 1200 cm^{-1}. **c) – d)** The topography **(c)** and the near-field amplitude image **(d)** of BP after being exposed to atmospheric conditions for 3 days. The insets in **a)** and **c)** show the topography detail of the black square with a 200 nm scale bar.

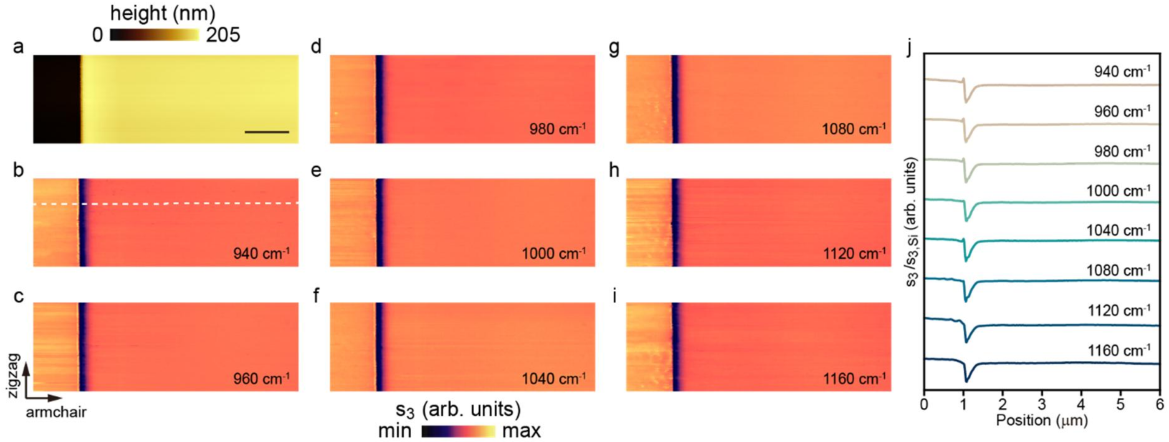

Supplementary Figure 12. Monochromatic near-field amplitude images of the pristine BP. a) The topographic of 205 nm thick BP. Scale bar: 1 μm. **b) – i)** Monochromatic near-field amplitude images of BP from 940 – 1160 cm^{-1}. **j)** Near-field profiles from position marked by the white dashed line in **(b)**.

Supplementary References

1 Asahina, H., Shindo, K. & Morita, A. Electronic Structure of Black Phosphorus in Self-Consistent Pseudopotential Approach. *Journal of the Physical Society of Japan* **51**, 1193-1199 (1982).

2 Ribeiro, H. B., Pimenta, M. A. & de Matos, C. J. S. Raman spectroscopy in black phosphorus. *Journal of Raman Spectroscopy* **49**, 76-90 (2018).

3 Beard, M. C., Turner, G. M. & Schmuttenmaer, C. A. Transient photoconductivity in GaAs as measured by time-resolved terahertz spectroscopy. *Physical Review B* **62**, 15764-15777 (2000).

4 Charnukha, A. *et al.* Ultrafast nonlocal collective dynamics of Kane plasmon-polaritons in a narrow-gap semiconductor. *Science Advances* **5**, eaau9956 (2019).

5 Wang, L. *et al.* Manipulating phonon polaritons in low loss 11B enriched hexagonal boron nitride with polarization control. *Nanoscale* **12**, 8188-8193 (2020).

6 Chi, L., Lee, M.-H., Chu, T.-Y. & Tao, Y. Excitonic effect in black phosphorus oxides. *2D Materials* **9**, 015007 (2021).

7 Zhou, Q., Chen, Q., Tong, Y. & Wang, J. Light-Induced Ambient Degradation of Few-Layer Black Phosphorus: Mechanism and Protection. *Angewandte Chemie International Edition* **55**, 11437-11441 (2016).

8 Dai, S. *et al.* Tunable Phonon Polaritons in Atomically Thin van der Waals Crystals of Boron Nitride. *Science* **343**, 1125-1129 (2014).

9 Asahina, H. & Morita, A. Band structure and optical properties of black phosphorus. *Journal of Physics C: Solid State Physics* **17**, 1839 (1984).

10 Lee, S.-Y. & Yee, K.-J. Black phosphorus phase retarder based on anisotropic refractive index dispersion. *2D Materials* **9**, 015020 (2022).

11 Sternbach, A. J. *et al.* Programmable hyperbolic polaritons in van der Waals semiconductors. *Science* **371**, 617 (2021).

12 Huber, M. A. *et al.* Femtosecond photo-switching of interface polaritons in black phosphorus heterostructures. *Nature Nanotechnology* **12**, 207-211 (2017).

13 Ni, G. X. *et al.* Ultrafast optical switching of infrared plasmon polaritons in high-mobility graphene. *Nature Photonics* **10**, 244-247 (2016).

14 Fei, Z. *et al.* Infrared Nanoscopy of Dirac Plasmons at the Graphene–SiO2 Interface. *Nano Letters* **11**, 4701-4705 (2011).

15 Wagner, M. *et al.* Ultrafast Dynamics of Surface Plasmons in InAs by Time-Resolved Infrared Nanospectroscopy. *Nano Letters* **14**, 4529-4534 (2014).

16 Favron, A. *et al.* Photooxidation and quantum confinement effects in exfoliated black phosphorus. *Nature Materials* **14**, 826-832 (2015).

17 Menabde, S. G. *et al.* Near-field probing of image phonon-polaritons in hexagonal boron nitride on gold crystals. *Science Advances* **8**, eabn0627 (2022).

18 Zheng, Z. *et al.* Highly Confined and Tunable Hyperbolic Phonon Polaritons in Van Der Waals Semiconducting Transition Metal Oxides. *Advanced Materials* **30**, 1705318 (2018).

19 Dai, S. *et al.* Phonon Polaritons in Monolayers of Hexagonal Boron Nitride. *Advanced Materials* **31**, 1806603 (2019).

20 Harrison, H. *et al.* Quantification of hexagonal boron nitride impurities in boron nitride

nanotubes via FTIR spectroscopy. *Nanoscale Advances* **1**, 1693-1701 (2019).
21 Dai, S. *et al.* Subdiffractional focusing and guiding of polaritonic rays in a natural hyperbolic material. *Nature Communications* **6**, 6963 (2015).
